# Resilience and Alternative Stable States of Tropical Forest Landscapes under Shifting Cultivation Regimes

**DOI:** 10.1371/journal.pone.0137497

**Published:** 2015-09-25

**Authors:** Piotr Magnuszewski, Katarzyna Ostasiewicz, Robin Chazdon, Carl Salk, Michal Pajak, Jan Sendzimir, Krister Andersson

**Affiliations:** 1 Risk, Policy and Vulnerability Program, International Institute for Applied Systems Analysis, Laxenburg, Austria; 2 Centre for Systems Solutions, Wroclaw, Poland; 3 Department of statistics, Wroclaw University Of Economics, Wroclaw, Poland; 4 Department of Ecology and Evolutionary Biology, University of Connecticut, Storrs, Connecticut, United States of America; 5 Institute of Behavioral Science, University of Colorado at Boulder, Boulder, Colorado, United States of America; 6 Ecosystem Services and Management Program, International Institute for Applied Systems Analysis, Laxenburg, Austria; 7 Southern Swedish Forest Research Center, Swedish University of Agricultural Sciences, Alnarp, Sweden; 8 Department of Mathematical Economics, Wroclaw University of Economics, Wroclaw, Poland; University of New South Wales, AUSTRALIA

## Abstract

Shifting cultivation is a traditional agricultural practice in most tropical regions of the world and has the potential to provide for human livelihoods while hosting substantial biodiversity. Little is known about the resilience of shifting cultivation to increasing agricultural demands on the landscape or to unexpected disturbances. To investigate these issues, we develop a simple social-ecological model and implement it with literature-derived ecological parameters for six shifting cultivation landscapes from three continents. Analyzing the model with the tools of dynamical systems analysis, we show that such landscapes exhibit two stable states, one characterized by high forest cover and agricultural productivity, and another with much lower values of these traits. For some combinations of agricultural pressure and ecological parameters both of these states can potentially exist, and the actual state of the forest depends critically on its historic state. In many cases, the landscapes’ ‘ecological resilience’, or amount of forest that could be destroyed without shifting out of the forested stability domain, declined substantially at lower levels of agricultural pressure than would lead to maximum productivity. A measure of ‘engineering resilience’, the recovery time from standardized disturbances, was independent of ecological resilience. These findings suggest that maximization of short-term agricultural output may have counterproductive impacts on the long-term productivity of shifting cultivation landscapes and the persistence of forested areas.

## Introduction

Reducing deforestation in tropical landscapes remains a major thrust of efforts to mitigate climate change and preserve biological diversity. Recent scientific work has warned that some tropical regions are approaching critical tipping points and may undergo sudden shifts to a new ecological equilibrium characterized by reduced carbon storage and biological diversity [1], [2], with forests replaced by low-diversity grasslands, a phenomenon that has been observed in Madagascar [3] and Bolivia [4]. Tropical agro-ecosystems and their surrounding landscapes face increased pressure from population growth, increased climate variability, inadequate protection of forest reserves, and in some places, loss of local ecological knowledge [5]. As a result of such stressors, an increasing number of ecosystem collapses are being observed in tropical regions [6], [7]. Despite this observed pattern, factors that may push tropical forest landscapes into deforested, low-productivity and low-diversity stable states remain poorly understood. This uncertainty hampers efforts to manage forested landscapes for human purposes while also maintaining their ecological functions and cultural capital. Here, we develop a coupled model of the interactions between human land-use decisions and tropical forest dynamics to evaluate how intensified shifting cultivation and reduction in post-fallow succession rates influence alternative stable states and the resilience of these states. We further investigate how tropical forest resilience is linked to agricultural productivity by examining conditions where increasing agricultural land-use pressure at the landscape scale can lead to the collapse of both resilient forest ecosystems as well as the coupled agricultural production system.

Shifting cultivation has been an important land management regime in tropical regions for millennia. Millions of rural smallholders continue to depend on shifting cultivation systems to fulfill their basic food needs [8]. A sustainable shifting cultivation system consists of rotating agricultural use of small plots in which forest is cut, burned, and farmed for 1–2 seasons, followed by forest regeneration, often actively assisted, during a fallow period until soil fertility recovers to support productive agriculture again. As traditionally practiced, shifting cultivation does not necessarily result in permanent or large-scale deforestation because the forest ecosystem is allowed to recover through natural regeneration for several years after a plot has been cleared and farmed. The salient question around shifting cultivation is the relative footprint of agriculture on the landscape and its resulting impact on biodiversity, carbon storage, and other landscape-level responses to a wide range of disturbances. Shifting cultivation plays a central role in the cultural production systems of many indigenous peoples [9], [10], [11], [12], [13], [14] and relies on sophisticated knowledge of succession, seed dispersal, soil processes, species characteristics, and species interactions in tropical forest ecosystems. For example, the Yucatec Maya of southern Mexico have practiced sustainable land use rotation for over 4,000 years and their language reveals detailed ecological knowledge with words for seven different successional stages and at least 25 different soil types [15]. Similar knowledge systems are observed in virtually all countries with tropical forests [3], [16], [17].

The sustainability of shifting cultivation relies upon the regenerative capacity of forests to maintain agricultural productivity, and is an example of social-ecological resilience, as both human societies and forests are sustained in the long term [18]. Currently, shifting cultivation is predominantly practiced by smallholders and is often mixed with other forms of land use and non-farm income, providing diverse livelihoods for rural families while maintaining regional biodiversity and ecosystem services. Growing populations, increasing access resulting from the new road construction, and governmental pressure to settle permanently and privatize communal lands have reduced the sustainability of shifting cultivation by shortening fallow periods and reducing landscape-level forest cover in many regions [19]. In some places, government policies favor intensive permanent agriculture and tree plantations, and actively discourage shifting cultivation [12], [20].

The dynamic balance between cultivation pressure and productivity in shifting-cultivation systems is poorly understood, hampering efforts to preserve ecological and cultural aspects of this potentially sustainable land-use system. Studies have clearly demonstrated, however, that the intensity and duration of previous land use [3] and the extent and diversity of landscape forest cover [21] affect the speed and trajectory of forest regrowth. These factors, in turn, affect soil fertility, food production, and human livelihoods in shifting cultivation systems. Premature clearing of successional forests, before soil fertility and aboveground biomass have substantially recovered, reduces agricultural yields and decreases future forest regenerative capacity [22], [19], [23]. If premature clearing occurs repeatedly, succession is arrested and forest does not spontaneously regenerate in areas previously cleared for agriculture. Arrested succession leads to alternative stable states dominated by shrubs or weedy grasses [3], [4]. Poor forest regeneration, in turn, feeds back to reduce agricultural productivity. Soil fertility is reduced, and fields become more susceptible to weed invasions and fire. The link between forest regeneration and recovery of soil fertility leads to further declines in forest regenerative capacity [24], [25], [26]. Thus, agricultural output and non-crop resources are directly linked to landscape-level forest condition.

Models of coupled social-ecological systems (e.g. forests, grasslands and lakes affected by human activities) can show abrupt, nonlinear shifts between alternative configurations [27], [28]. The practical significance of alternative stability domains is seen when a system undergoes a transition that is difficult or impossible to reverse [29]. Although regime shifts are seen in many stylized models, demonstrating whether and how such phenomena might occur in the real world is not simple. In spite of methodological and practical difficulties, evidence for the existence of alternative stability domains has been found in a few specific ecosystems and socio-ecological systems [30], [31], [32], [33], [34]. Hirota et al. [35] provide evidence that in many places forest and savanna are alternative stable states with precipitation-driven feedbacks, although this relationship is also affected by climate change, deforestation, changing fire regimes, and other human-influenced factors [36]. Experimental manipulations are intractable in most types of ecosystems with the notable exception of lake microbial and fish communities in which alternative stable states have been directly demonstrated [37], [38], [39].

Descriptive case studies of shifting-cultivation systems suggest that given a set of ecological conditions (soil fertility, seasonality of rainfall, etc.) there is a ‘tipping point’ of agricultural pressure that leads to the loss of forest regeneration capacity within a shifting-cultivation landscape [19]. For the first time, we model this process quantitatively, and demonstrate how land-use decisions affect the resilience of the forested states at the landscape level. We develop and test two hypotheses using our model and parameters based on published case studies. First, we hypothesize that our coupled forest-agricultural model will exhibit two stable states: one of them characterized by abundant intermediate- and advanced-stage successional forest and high agricultural productivity and the second with no forest regeneration beyond early-successional scrub. Second, we hypothesize that maximizing agricultural productivity reduces resilience of the forested state, increasing the risk of transition to a deforested state. Our analyses cover resilience in both the ecological sense (how big of a disturbance the system can endure without shifting to an alternative state) and the engineering sense (how long the system requires to recover from a disturbance) [40].

## Methods

### The forest landscape model

**Fig 1:**
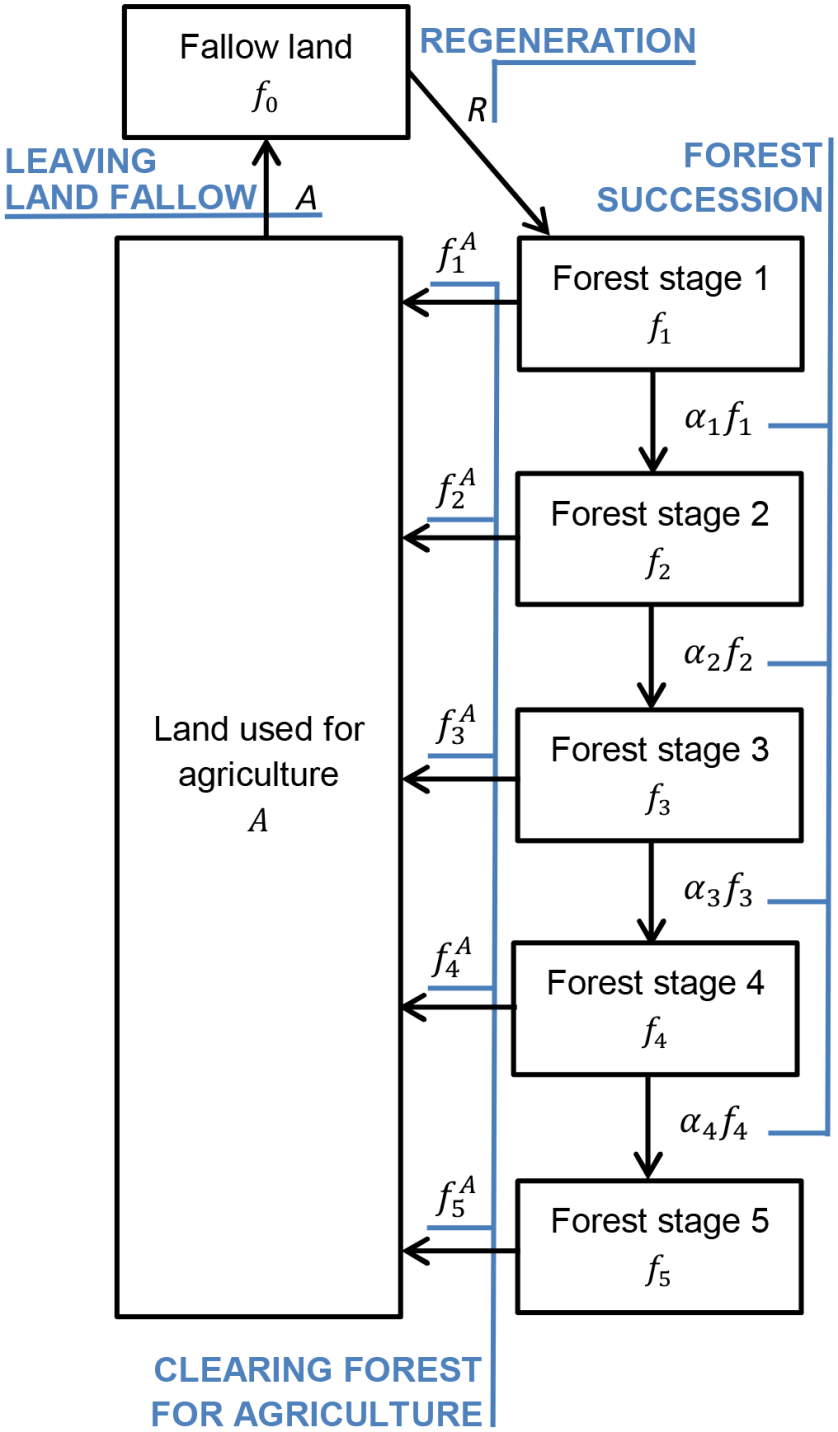
A graphical representation of the forest-landscape model used in this paper. See table 1 for additional explanation of symbols. Note that under the model, land used for agriculture becomes unproductive and must be left fallow during the next time step, so both of these values are indicated with the same symbol.

We test our hypotheses using a dynamical systems approach that incorporates feedbacks between deforested and forested components of a simple model of a shifting-cultivation landscape (Fig 1). In our model, landscape-level forest condition depends only on the balance between forest regeneration—the succession of agricultural fallows into different forest stages—and forest clearing for cultivation. After a period of agricultural use, fallow patches gradually regenerate into forest through a series of successional stages, as long as they are not re-cleared in the interim. Crop productivity is higher on land converted from more advanced forest stages (stages 3–5 in Fig 1). The fallow regeneration rate increases with landscape-level forest condition, defined by the amount of forest cover in intermediate and advanced successional stages. Model outputs include landscape-level forest condition and agricultural output. We assume a best-case scenario in which forest regeneration is not impeded by invasive species, effects of climate change, defaunation, or by harvesting for timber or non-timber products. However, we perform a series of simulations of responses to catastrophic disturbances. We also assume that cultivation is spread across the landscape in such a way that seed dispersal does not limit succession, eliminating the need for a computationally-intensive, spatially-explicit model. We parameterize this model using published field studies from Mexico [15], Borneo [16] and Madagascar [3] (See Supporting Information for more details).

**Table 1:** Brief explanations of symbols used for variables and parameters in this paper.

Symbol	Variable/Parameter	Type/Use	Range of values and type of unit
*A*	Area of forest cleared for agriculture	State variable	〈0,*A*_*p*_〉, patches
*A*_*i*_	Area of land in stage *i* cleared for agriculture	Variable	〈0,*A*_*p*_〉, patches
*A*_*p*_	Agricultural pressure	Analyzed parameter	〈0,*F*〉, patches
*α*_*i*_	Transition rate from forest of stage *i* to forest of stage *i*+1	Analyzed parameter	〈0,1〉, dimensionless
*β*_*i*_	Agricultural productivity coefficient for land converted from stage *i*	Analyzed parameter	≥0, product/patch
*F*_*c*_	Forest condition	Derived variable (variable computed from state variables)	≥0, patches*forest state
*f*_0_	Fallow land	State variable	〈0,100〉 patches
*f*_*i*_	Forest of stage *i*	State variable	〈0,100〉, patches
{*f*}_*s*_	Forest stable state	Vector of values of forest stages corresponding to a stable state	Each individual value has potential range of 〈0,100〉 with units of patches
*γ*_*i*_	Structural and reproductive development of stage *i*	Fixed parameter (values are not changed during model runs or model analysis)	≥0, forest state
*P*	Agricultural production	Variable	≥0, product
*P*_*i*_	Agricultural production from land converted from forest of stage *i*	Variable	≥0, product
*R*	Forest recruited from fallow land.	Variable	〈0,*f*_0_〉, patches
*r*	Local recruitment rate	Analyzed parameter	≥0, 1/forest state
*r*_0_	External recruitment rate	Fixed parameter	≥0, patches
*ρ*	Ecological resilience of the forested landscape	Derived variable	〈0,1〉, proportion of patches
*T*_0_	Recovery time in the absence of agricultural pressure	Derived variable	≥0, time
*T*_*R*_	Engineering resilience (recovery time following a standardized disturbance)	Derived variable	≥0, time
*T*_*R*_/*T*_0_	Relative recovery time	Derived variable	≥0, dimensionless

Central to our model is a landscape with 100 patches that are represented non-spatially. Forest is cleared for agriculture. Agricultural land use (*A*) is also referred to as agricultural clearing. After abandonment, land enters a fallow stage (*f*_0_) that cannot support crop production, which undergoes regeneration along a successional pathway of up to five stages ranging from young scrub to old-growth (Fig 1; *f*_1_ to *f*_5_) if sufficient time passes without re-clearing. In each annual time step forest patches transition into the next successional stage *F*_n+1_ with a transition rate *α*_*n*_, equivalent to a patch of forest persisting in stage *n* for an average of *r* = 0.5 years.

The landscape-wide state of the forest is measured by a forest condition variable (*F*_*c*_), which feeds back to influence forest regeneration and productive agricultural use. Forest condition (*F*_*c*_) is the sum of the land in each of the five forest stages, weighted by the area in each stage:

$$F_{c}=\gamma_{1}f_{1}+\gamma_{2}f_{2}+\gamma_{3}f_{3}+\gamma_{4}f_{4}+\gamma_{5}f_{5}$$ (1)

Fallow land (*f*_0_) has a weight of 0, so does not contribute to the overall calculation of forest condition (*F*_*c*_). The weights *γ*_*i*_ indicate how each forest stage promotes the regeneration of early successional forest throughout the landscape.

In our model, the forest recruited from fallow land (*R*) to the earliest stage forest is a function of colonization and establishment from forest outside the modeled landscape (external recruitment rate; *r*_0_) and inside the modeled landscape (local recruitment rate *rF*_*c*_). The local recruitment rate *rF*_*c*_ is the product of forest condition (*F*_*c*_) and a constant local recruitment rate (*r*):

$$R=r_{0}+rF_{c}$$ (2)

We used a single value for the external recruitment rate (*r*_0_) in our analysis.

### Agricultural productivity model

Clearing for agriculture (*A*) is equal to the agricultural pressure (*A*_*p*_) or the amount of land available for conversion, whichever is smaller. As soil fertility fully recovers by successional stage 3, forests from stages 3, 4 and 5 are preferred, in that order, for clearing, followed by less fertile stages 2 and 1. This order is based on a preference for nutrient-rich soils and increasing difficulty of clearing the oldest forests for farming. All trees are removed from these areas followed by conversion into agricultural plots. Agricultural production from each plot depends on the stage of the forest that was cut to create it. The area converted into agriculture that originated from the forest stage *f*_*i*_ is denoted by $f_{i}{}^{A}$, as illustrated in Fig 1. Cleared land remains in cultivation for only one time step and subsequently becomes fallow land. Only forested land (not early fallows) can be converted for agriculture.

The main model outputs are landscape-wide forest condition *F*_*c*_ (see Eq 1 and description above) and agricultural productivity *P*. The model does not consider harvest of timber and non-timber forest products, as the focus of our study is the balance between agricultural pressure and productivity. Although real forests provide various products that when consumed or sold can substitute for crops and reduce agricultural demand, including such effects in our model would slightly shift equilibria without changing the overall dynamics. The landscape-level agricultural productivity, *P*, is given by the sum of production from all stages that were cut:

$$P=\sum_{i=1}^{5}\beta_{i}(f_{i}\to A)$$ (3)

where *β*_*i*_, *i* = 1…5 are agricultural productivity coefficients based on successional stage-specific soil fertilities. The full set of model equations is presented in the Supporting Information. We use the analytical methods of dynamical systems to identify stable states’ existence, number and dependence on model parameters. Such structural analysis is often more revealing than analysis of model outputs for specific sets of parameters as it allows us to understand the range of possible system behaviors.

### Ecological resilience of the forest-agricultural system to external disturbances

Agricultural pressure not only leads to forest removal but also decreases the resilience of the forested stable state, making it more vulnerable to external disturbances that can further reduce forest cover. To quantify the ecological resilience of the modeled landscapes, we apply external shocks to the system as described below. Such disturbances could be natural or anthropogenic, including hurricanes, fires, or conversion of forests to pasture. To preserve the general character of our analysis we assume that each of the forest stages is equally affected by this external disturbance.

The resilience of a stable forested state {*f*}_*s*_ that has specific quantities of forest in each stage {*f*_1_,*f*_2_,*f*_3_,*f*_4_,*f*_5_}, can be defined with respect to a sudden external disturbance (shock) that occurs within a single time-step that destroys (i.e. turns into *f*_0_) an equal fraction of all forest stages. After the shock, the forest is in the new state: {(1-*x*)*f*_1_,(1-*x*)*f*_2_,(1-*x*)*f*_3_,(1-*x*)*f*_4_,(1-*x*)*f*_5_}, where *x*∈〈0,1〉 denotes the fraction of forest destroyed. This new state is in most cases not a stable state, so the system will evolve to one of the stable states over time. The resilience of a forested stable state is defined as the maximal fraction of the forest that can be destroyed with the system returning to the forested state:

$$\rho =\text{max}(x):(1-x){\left\{f\right\}}_{s}\to {\left\{f\right\}}_{s}$$ (4)

where the arrow denotes transition to a stable state and *x* is limited to values in the interval [0,1].

### Forest Recovery Time—Engineering Resilience

The measure of ecological resilience considered above reveals how much perturbation a forested landscape can take and still return to the forested stable state. However, it does not tell us how much time it takes to return to this state; this value is the system’s ‘engineering resilience’ [40]. The distinction between ecological and engineering resilience is important because in a real ecosystem, additional perturbations or an increase in agricultural pressure can happen during the recovery period, increasing the risk of pushing the system into the degraded stable state.

To investigate the recovery time, *T*_*R*_, we employ a standardized perturbation of 5% destruction of each forest stage and measure how many time steps are required for the forest to return to within 0.1% of its pre-perturbation state (using a margin of 1% gives very similar results). With this standardized perturbation, we can measure engineering resilience for any landscape with an *ecological* resilience of at least 5%, that is to say any landscape that can suffer 5% forest loss without shifting to the deforested state. During this simulation, agricultural pressure (*A*) is left unchanged. We also investigate the recovery time of fully mature forest (*f*_5_ = 100 and f_1−4_ = 0 *f*_1−4_ = 0; this occurs only for *A* = 0), not only for the ‘standard perturbation‘ defined above (and equal to 5% of the forest condition) but also for some more extreme perturbations (note that because agricultural pressure is zero (*A* = 0) the deforested stable state does not exist allowing us to investigate all sizes of perturbations up to 100%)

For engineering resilience calculations, we will focus on forest states for which the most mature stage (*f*_5_) still exists, that is, for *f*_5_>0. There are two reasons for this restriction: 1) this is the most interesting case as such forests are of high conservation value, and 2) when *f*_5_>0 return times become very short—a few time-steps—which may lead to artifacts due to the discrete representation of time in our model. Moreover, it turns out that for all cases considered in this article, for *f*_5_>0 ecological resilience is always 100% (although in general this doesn’t have to be true). That is, if any *f*_5_ stage forest exists, even a very small amount, the forest—even after complete destruction—will eventually recover to the initial state if there are no further perturbations or change in agricultural pressure. However, the important issue with respect to engineering resilience is how much time is required for this recovery.

## Results

### Existence of alternative stable states

Depending on the values of agricultural pressure (*A*_*p*_) and the local recruitment rate (*r*), the model predicts a landscape with one or two alternative stable states. The first of these states is characterized by low-productivity agriculture that we call the ‘deforested‘ state. This state consists only of the youngest forest stage (*f*_1_), fallow (*f*_0_), and agricultural land (*A*). The other state, which we call the “forested” state, exists at low to moderate levels of agricultural pressure and features a landscape with substantial late successional forest. Detailed closed-form solutions for these stable states are provided in the Supporting Information.

**Fig 2:**
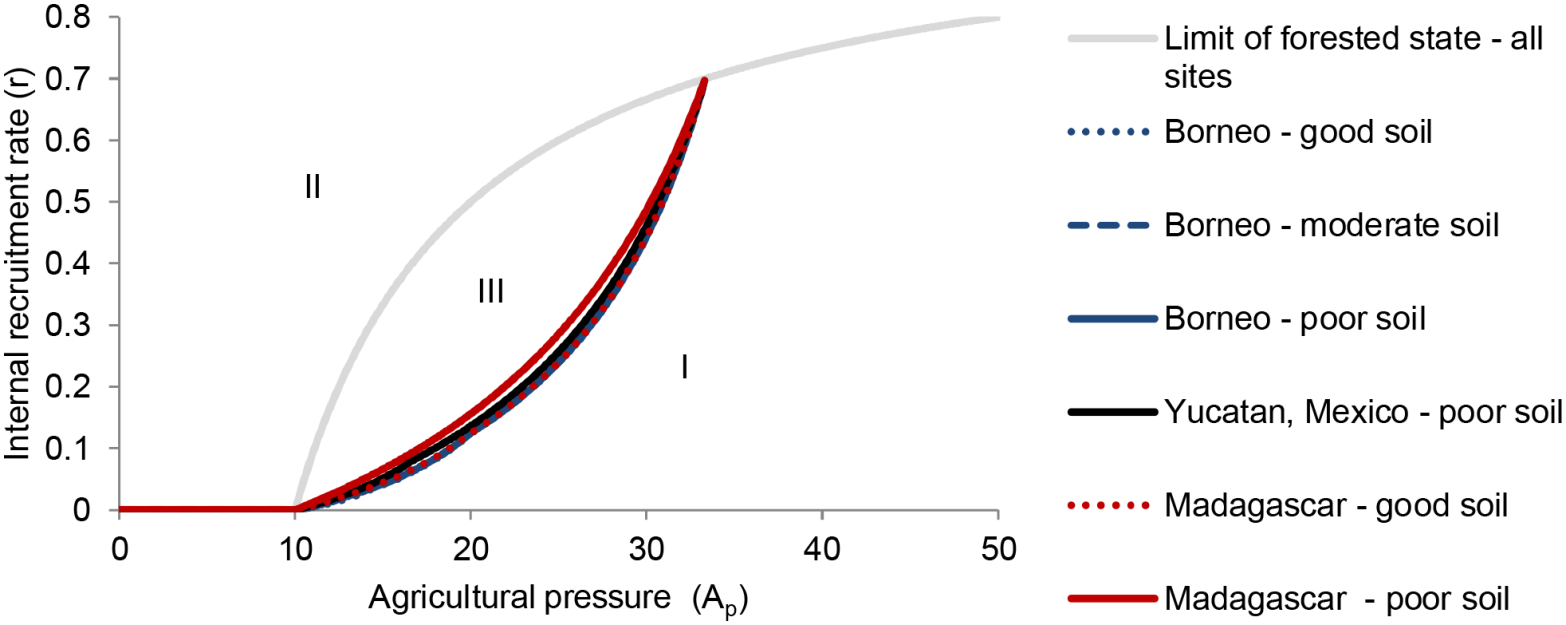
Stable states of the modeled shifting cultivation system. The stable states are shown here as a function of agricultural pressure and local forest recruitment rate. Region I has little forest cover (deforested stable state) while region II has extensive forest cover (forested stable state). In region III either the forested or deforested state is possible, depending on the previous forest and soil condition. The existence of two alternative stable states in this part of the model’s parameter space results in a hysteresis such that the current state of the system has two possible configurations depending on its previous state [30].

For various combinations of *A* and *r* (regions I and II in Fig 2), only one of the stable states exists. The regions of parameter space supporting each stable state are minimally sensitive to different transition rates among forest successional stages (*α*_*i*_) as parameter values from the different case studies yield very similar results (Fig 2). For the deforested state to occur *A*_*p*_ must be sufficiently high for a given value of r (shown by region I in Fig 2). In contrast, the forested state requires that *A*_*p*_ be sufficiently low for a given value of *r* (shown by regions II in Fig 2). For certain combinations of *A* and *r* both the deforested and forested states are possible (region III in Fig 2). In this situation, the state of the system depends on its past state. In other words, proportions of forest in different stages projected by the model depend on the initial proportions of forest in different stages. The lines separating regions I and III in Fig 2 are the limit of the forested state. They are slightly different among the six cases because within the forested state the proportion of land in different successional stages depends on *α*_*i*_. The line between regions II and III indicates the limit of the deforested state and is identical for all cases as the proportion of successional stages is independent of *α*_*i*_ in the deforested state. It is important to note that when agriculture pressure is low, only the ‘forested’ stable state is possible. At intermediate levels of agriculture pressure (the exact range depends on the specific parameter values) either stable state is possible. Finally, for high agriculture pressures (again, on the exact limit depends on the site-specific parameters) only the ‘deforested’ state exists.

**Fig 3:**
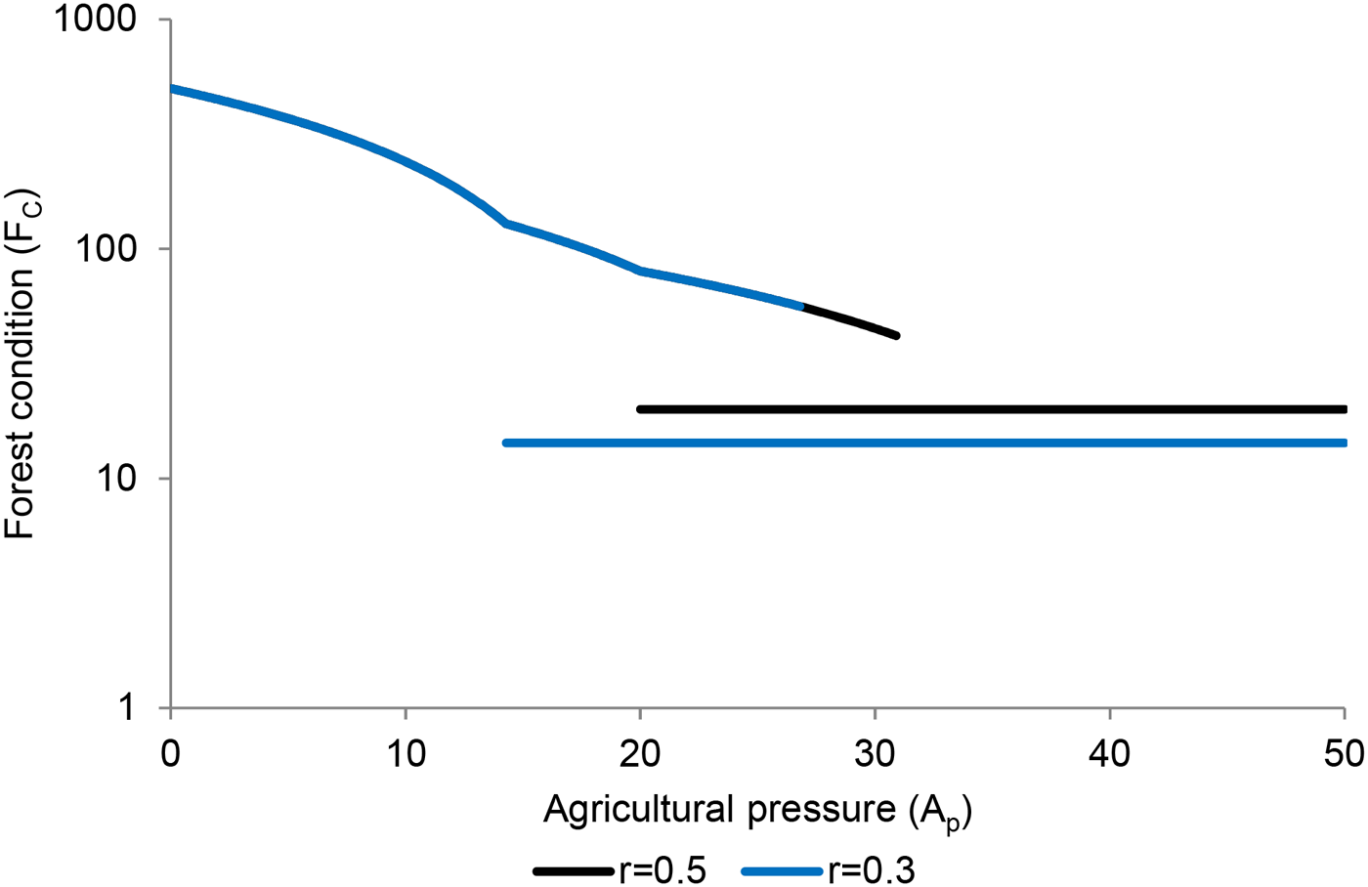
Forest condition as a function of agricultural pressure. The lines correspond to two different internal recruitment rates (*r* = 0.3 and *r* = 0.5). The diagonal lines correspond to the forested stable state and the horizontal lines the deforested state. Note that in the forested state, the lines for the two values of *r* overlap exactly for most values of agricultural pressure. For intermediate values of *A*_*P*_ two values of forest condition (*F*_*C*_) are possible due to hysteresis in the system. These results are from the Borneo (good soil) case.

Forest condition depends on the agricultural pressure and internal recruitment rate, and in some situations whether the system is in the forested or deforested stable state (Fig 3). Within the deforested state, forest condition depends only on internal recruitment rate. Within the forested state, forest condition depends on agricultural pressure, but not on internal recruitment rate. However, it is important to note the hysteresis effect described above: for intermediate levels of agricultural pressure, the socio-ecological system may be in either state, with big consequences for forest condition (Fig 3).

**Fig 4:**
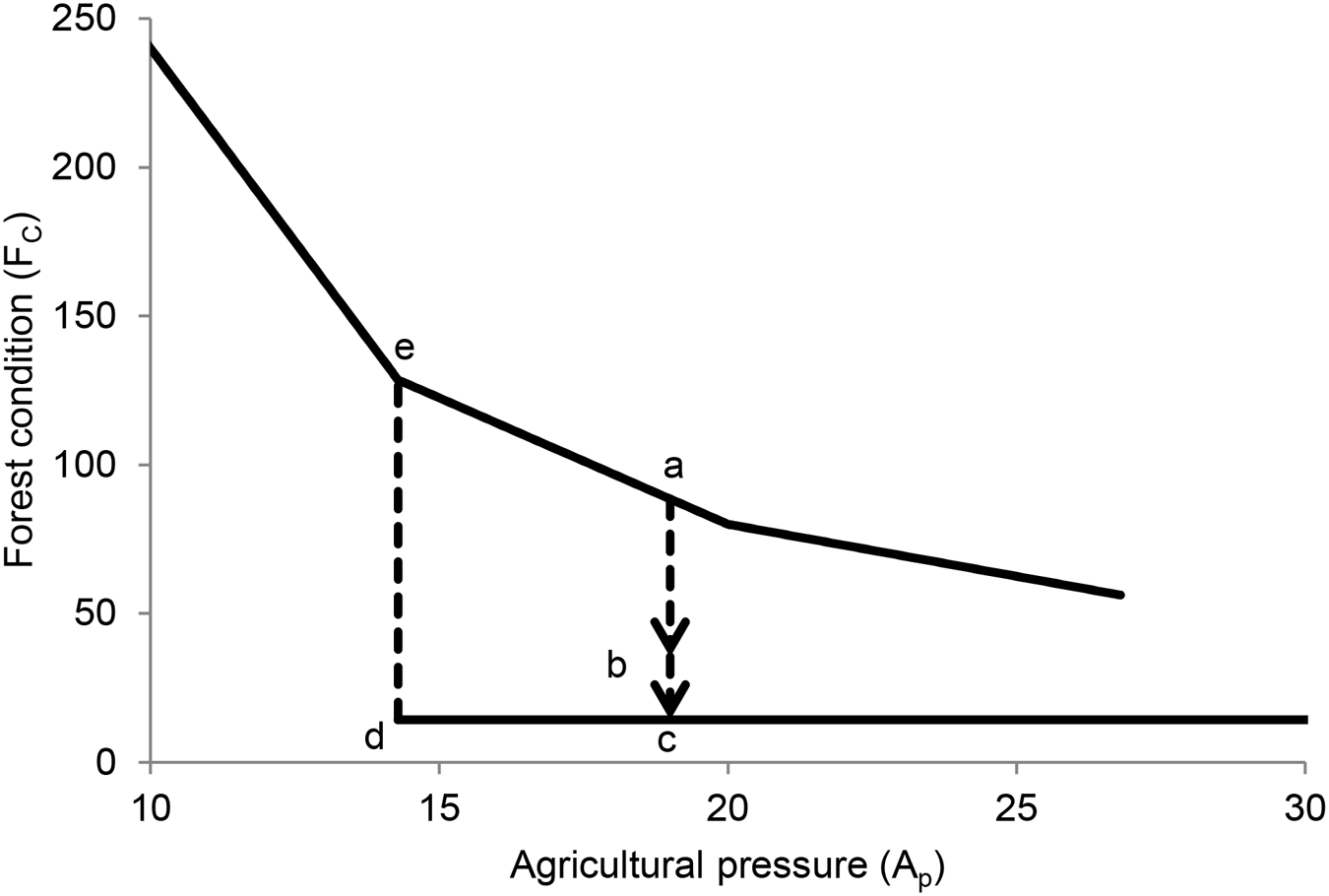
An example of a system transition between stable states. This example is based on the Borneo (good soil) case with internal recruitment rate *r* = 0.3. Point ‘a’ is the initial system state; point ‘b’ is the state following a disturbance, which leads to a forest collapse to point ‘c’ as agricultural pressure has not changed. Only by reducing agricultural demand to point ‘d’ may the system evolve back to the forested state (point ‘e’) when agriculture may safely be increased while maintaining the system in the forested state. See main text for additional details.

How this hysteresis plays out can be seen in Fig 4. In this example, the forest is initially in the state marked ‘a’ with *A* = 19 and forest condition *F*_*C*_ = 88.5. A disaster takes place that destroys 70% of the existing forest (point b). After that event, the same level of agricultural pressure leads to a forest collapse to state (c), with *F*_*C*_ = 14.29. After that collapse, the forest cannot recover unless agricultural pressure decreases at least to *A* = 14.29 (point d). At that value, the system will shift toward point ‘e’ at which point agriculture may be increased while maintaining substantial forest cover.

**Fig 5:**
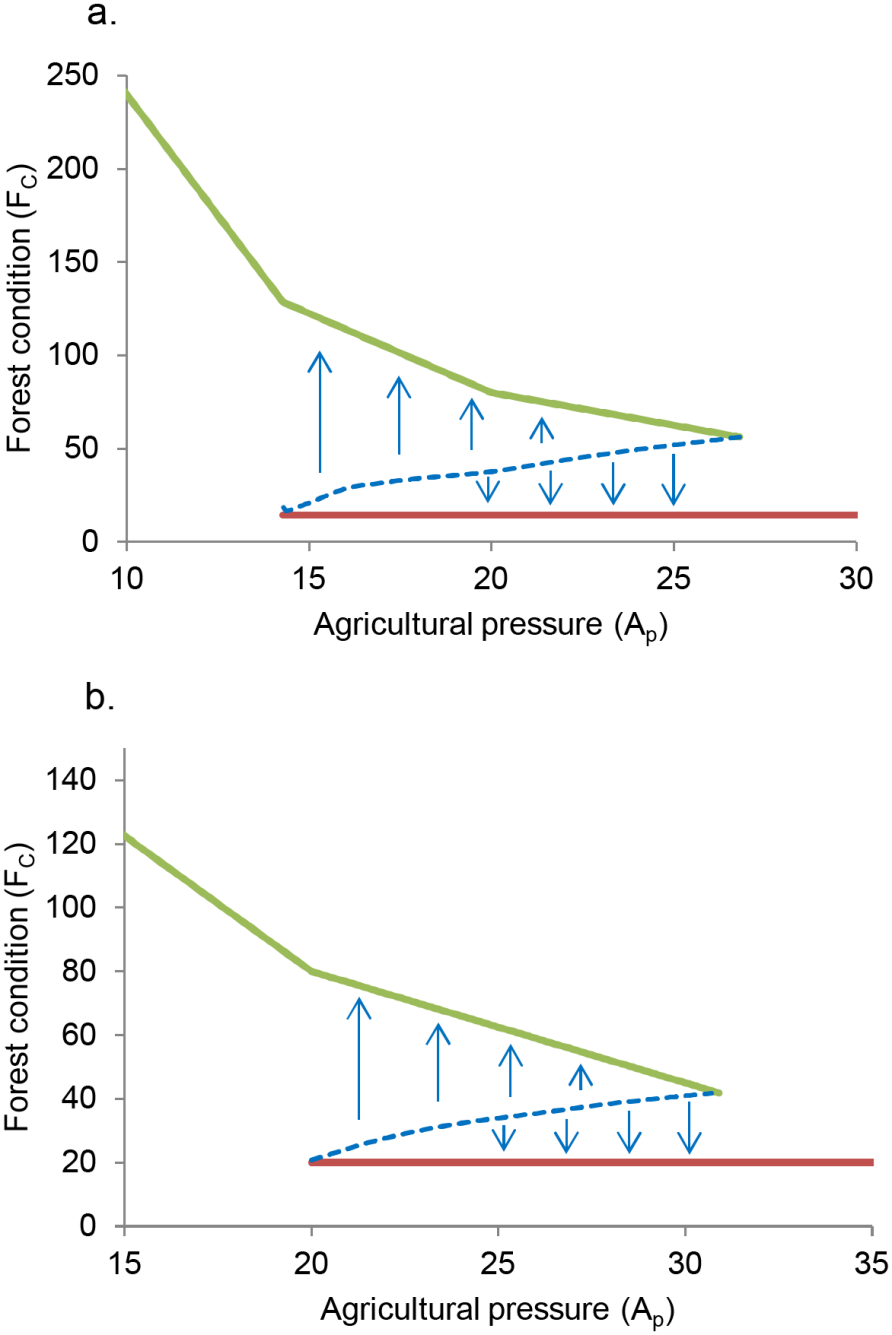
Basins of attraction of the forested and deforested stable states. Data shown are for the Borneo (good soil) case, for internal recruitment rates *r* = 0.3 (a) and *r* = 0.5(b).

The boundary between the stable states for values of agricultural pressure at which both states are possible is depicted in Fig 5. From this information it is possible to visualize potential shocks that may push the system into an alternative stable state. These ‘tipping points’ are strictly connected with the resilience of the system.

### Ecological Resilience of the forested stable state

**Fig 6:**
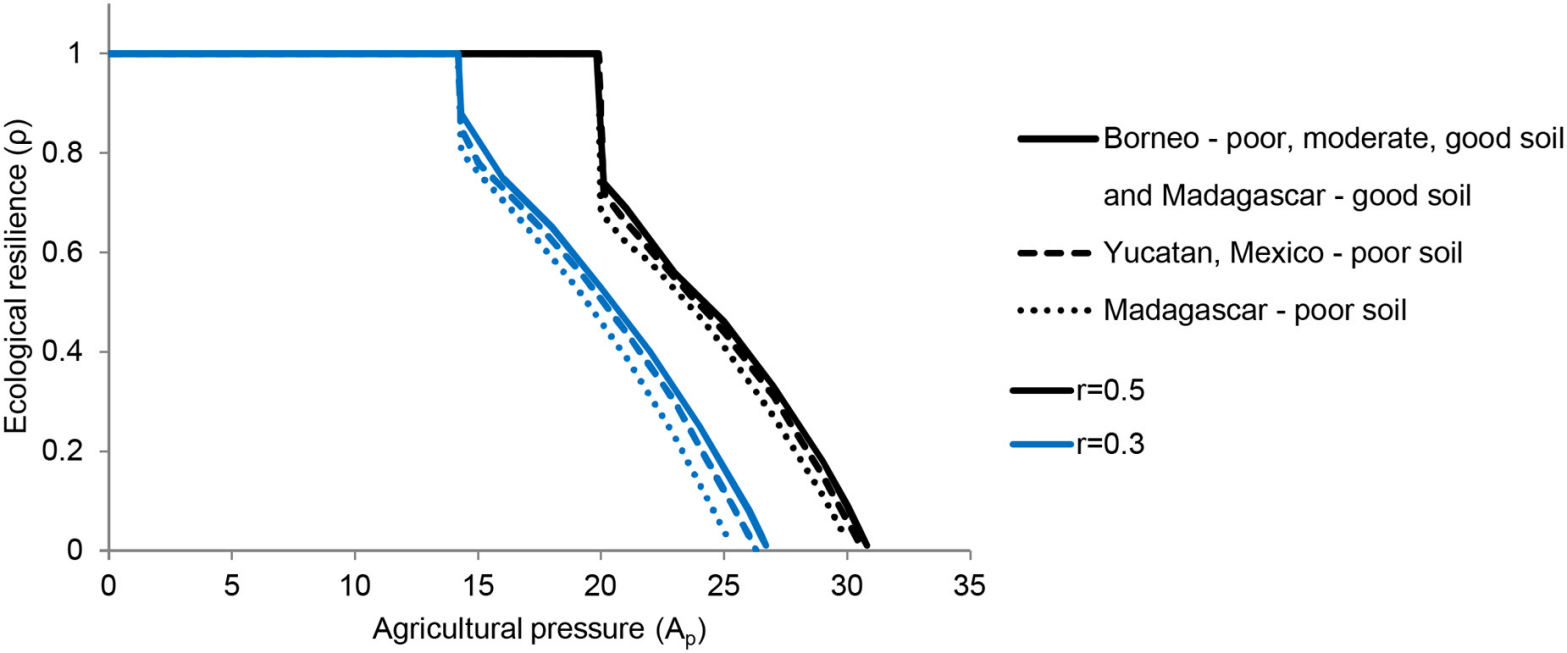
Ecological resilience of the forested stable state as a function of agricultural pressure. The ‘ecological resilience’ is the proportion of forested land that can be converted to agriculture without pushing the socio-ecological system into the deforested stable state. Results are shown for two different values (0.3 and 0.5) of the local recruitment rate parameter (*r*).

At low levels of agricultural pressure, ecological resilience of the landscape is 100% (Fig 6), since the forested state is the only possible equilibrium (Fig 2). With increasing agricultural pressure the deforested state becomes possible and the gap between the two states shrinks, leading to decreased resilience of the forested state. It reaches zero when agricultural pressure is sufficiently high that the forested state cannot be achieved (Fig 6). The forested state’s existence and ecological resilience depend heavily on the local recruitment rate in all cases (Fig 6). Despite differences in the modeled state of the forest under the different sites’ parameters, the resilience of the forested state differs only slightly among the case studies, assuming local recruitment rate is held constant across sites (Fig 6).

### Forest Recovery Time (Engineering Resilience)

**Fig 7:**
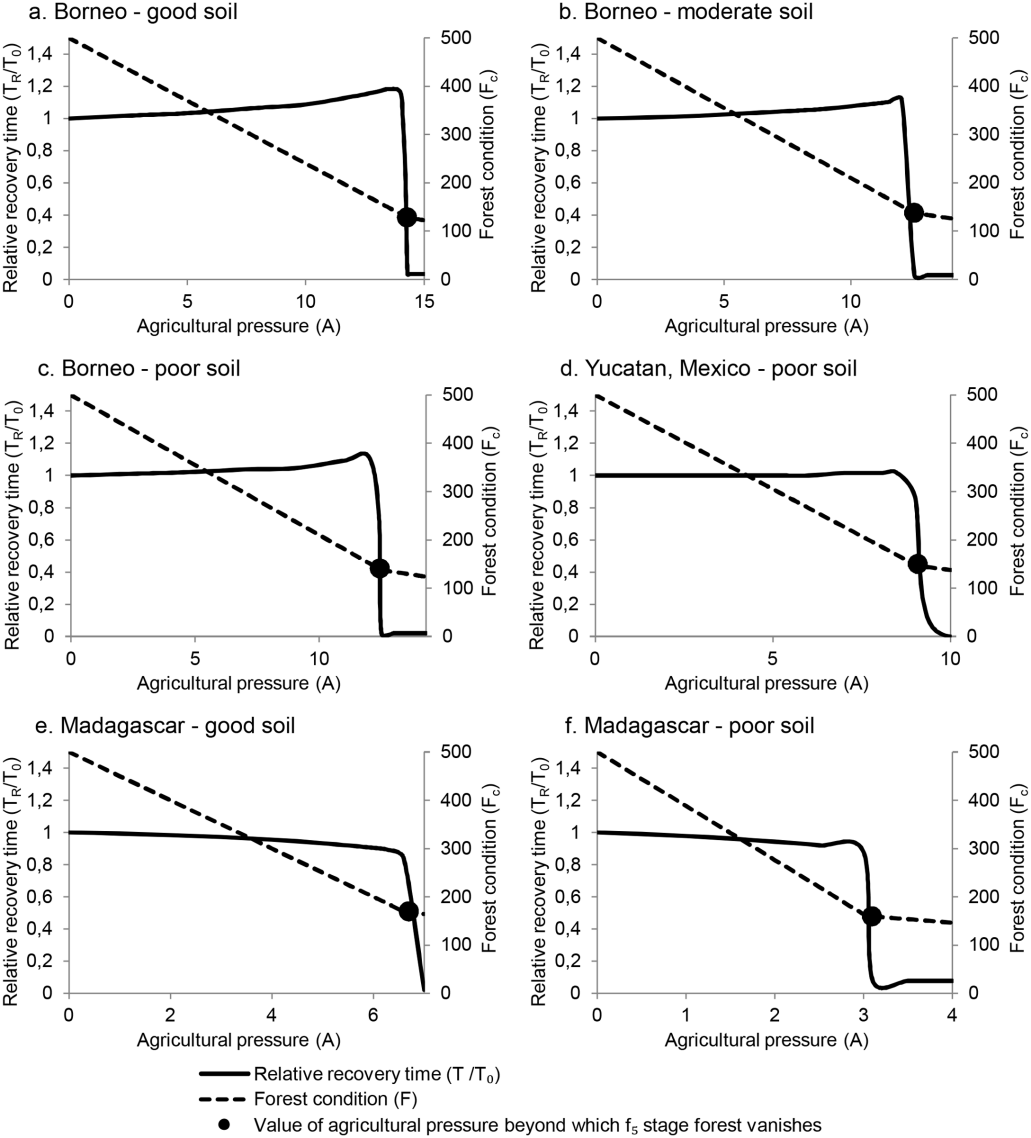
Relative recovery time following a standardized disturbance as a function of agricultural pressure. Note that this value is not an absolute number of time steps as in our definition of engineering resilience, but is scaled to the recovery time in the absence of agricultural pressure for easy comparison among the cases. The level of agricultural pressure at which *f*_5_ stage forest is no longer possible depends on the values of the forest recruitment rate and succession parameters. These plots are for a local recruitment rate (*r*) of 0.3; plots for *r* = 0.5 were virtually identical.

Recovery time to reach the pre-disturbance state as a function of agricultural pressure shows two contrasting patterns (Fig 7). For the three Borneo cases and the Yucatan case, recovery time increases gently with rising agricultural pressure until it reaches a critical point beyond which it abruptly decreases. However, in the Madagascar cases, recovery time decreases slightly with increased agricultural pressure, but still drops suddenly when high agricultural pressures are reached. Two opposing effects bring about these patterns. On one hand, forest state necessarily decreases with agricultural pressure. Thus, a standardized perturbation of 5% forest loss is a smaller absolute loss when agricultural pressure is higher. This means the absolute difference between the pre- and post-disturbance states is smaller, so recovery is faster. On the other hand, regeneration of fallow land to forest is proportional to the overall forest condition. This leads to slower regeneration at decreased forest states corresponding to higher agricultural pressure. Which of these two effects prevails depends on the specific values of the forest succession parameters (which are based on local soil factors) and the recruitment rate.

**Fig 8:**
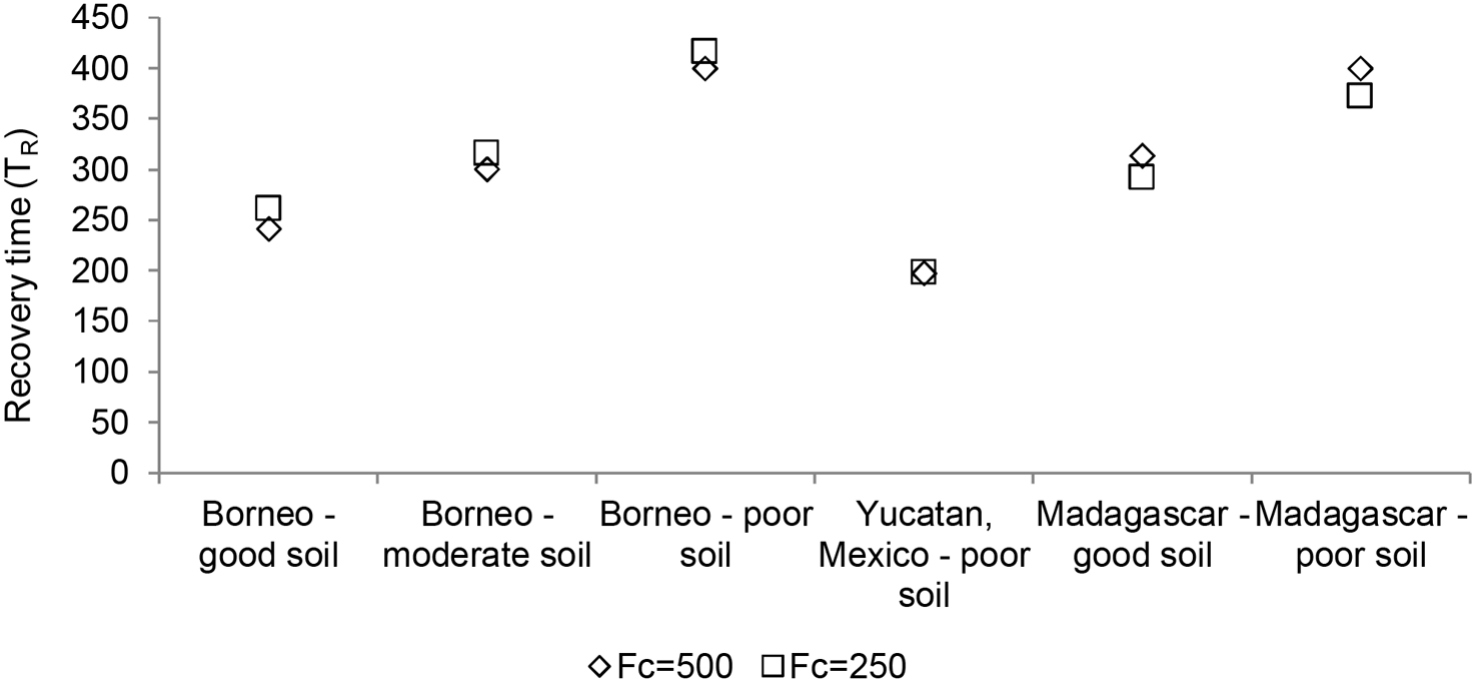
Comparison of recovery times following a standardized disturbance from two forest state starting points. The first starting point is a fully forested landscape (*F*_*c*_ = 500) which, by definition, has no agricultural pressure; the second is a landscape where the forest condition is at half its maximum possible value (*F*_*c*_ = 250). The level of agricultural pressure in the *F*_*c*_ = 250 case depends on the specific parameter values that result in this forest state. The values of *A* corresponding to this forest state can be seen in Fig 7.

**Fig 9:**
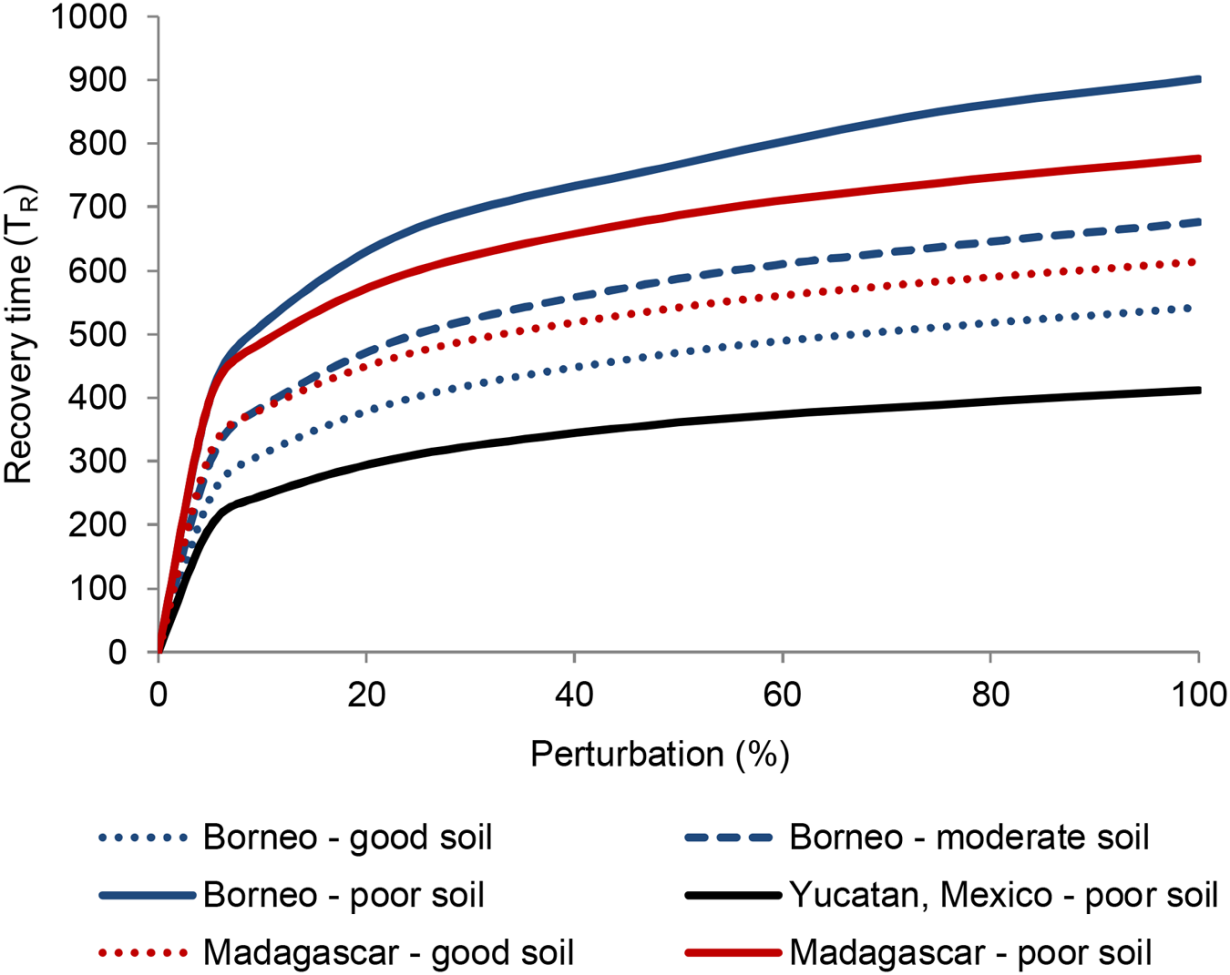
Recovery time of a fully mature forest as a function of the extent of perturbation. The fully mature forest state means that all patches are in the *f*_5_ stage, which, by definition, means there is no agricultural pressure.

The post-disturbance recovery time *T*_*R*_ is only weakly linked to ecological resilience. States of identical ecological resilience were found whose engineering resilience (recovery time) differed greatly. Within the range of agricultural pressure (A) for which ecological resilience is 100% (ρ = 1), a large range of recovery times is observed; moreover, big drops in *T*_*R*_ occur with only tiny increases in agriculture pressure near the point where the oldest (*f*_*5*_) stage of the forest vanishes (Fig 7). Further, recovery time is almost completely independent of the fallow to forest recruitment rate (*r*). Only under extremely high agricultural pressure (when ecological resilience of the forested state reaches zero) does *r* affect recovery time. Finally, the overall forest condition is only a weak predictor of recovery time (Fig 8). The same forest condition may show quite different recovery times depending on values of the forest succession parameters *α*_*i*_. Recovery time increases rapidly within the range of small disturbances (below about 10%), with further increases bringing smaller increases in recovery time (Fig 9).

### Agricultural Productivity

**Fig 10:**
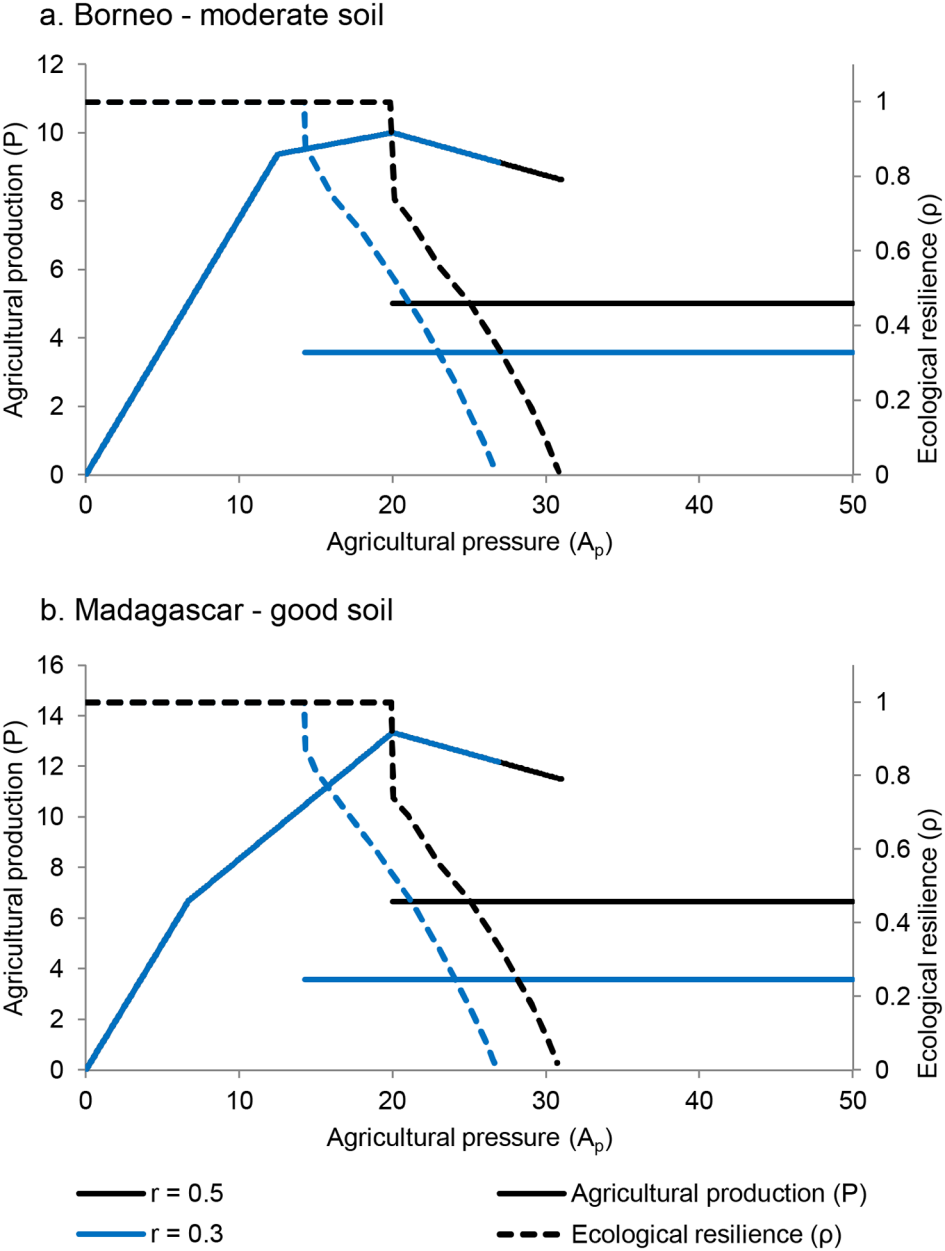
Agricultural production and ecological resilience of the landscape as a function of agricultural pressure. Two cases are presented: (a) Borneo—moderate soil and (b) Madagascar—good soil. Results are shown for two values of the local recruitment rate: *r* = 0.3 and *r* = 0.5. Most values on the upper branch of the agricultural production function are the same regardless of the value of local recruitment rate.

Agricultural output reaches a maximum at intermediate values of agricultural pressure (*A*_*p*_; Fig 10). The particular value of *A*_*p*_ at which this maximum is reached depends on the specific values of *α*_*i*_. Beyond this value, agricultural production decreases in most cases (Fig 10; Full derivation of these results is given in the Supporting Information). However, maintaining agricultural land use at this optimum carries risks for long-term agricultural output. This is because ecological resilience of the forested landscape decreases with increasing agricultural pressure, increasing the risk of a chance event pushing the system into the deforested state (Fig 10). For example, for the Borneo case with moderate soil (Fig 10a), when r_1_ = 0.3 *r* = 0.3 the agricultural pressure value leading to maximum production also reduces resilience by 50%. The risk of flipping to the deforested state counterbalances optimal agricultural output.

## Discussion

Based on a simple, empirically-grounded, set of assumptions about forest regrowth and recovery of soil fertility during succession, our model demonstrates that alternative stability domains are an inherent feature of shifting cultivation systems (Fig 2). Moreover, clearing more forest to maximize agricultural production results in a loss of resilience of the forested landscape, supporting our second hypothesis (Fig 6). Under these conditions, even if agricultural pressure remains constant over time, disturbances such as severe drought or a hurricane may suddenly push the predominantly forested landscape into a state of decreased forest cover and crop productivity that is not easily reversed. Furthermore, existence of alternative stability domains persists across the range of forest regeneration rates tested based on the case studies (Fig 2). Previous field- and model-based research has shown that mechanisms such as fire [41], or hydrological feedbacks [42] can create tipping points between an agro-forest landscape with a substantial forest component, and a deforested, low-productivity landscape. Our model shows that such outcomes can arise in the sole presence of agricultural pressure; the model’s two stable states result only from its internal dynamics. As we have modeled only the processes of forest regeneration and shifting cultivation, the results represent the basic processes underlying such systems. Our results present a best-case scenario of tropical landscape management as our model did not include additional factors that can negatively affect tropical forest regeneration such as elimination of seed dispersers [43], tree harvesting, or soil erosion [44]. Including these features and others such as seasonality which differ greatly among the case study regions would only increase the likelihood of a shift to the deforested state [45].

Our results are in broad agreement with field-based observations and historical narratives of shifting cultivation systems. Resilience of the forested landscape appears to decline with increasing agricultural pressure, as evidenced by increased fallow recovery times with additional cropping cycles [3], [19], [23]. However, to our knowledge, this process has not been explicitly modeled in previous work on shifting cultivation systems. Modeling the resilience of forest landscapes to human and natural disturbances provides important insights into the system’s dynamics because it is extremely difficult to empirically detect the proximity of a system to regime shift thresholds [46].

These findings have implications for policy making and land management where populations are growing and agriculture is intensifying. Although our modeling effort relies on a simple representation of the forest and agricultural dynamics, it provides important insights into sustainability of shifting cultivation systems. Because of its parsimony, our model is sufficiently flexible to be extended to show how forested landscape resilience may be affected by other disturbances such as logging, climate change, and invasive species. Our model provides a strong foundation for developing more complex landscape-level models that assess resilience of the forested state to other types of land-use practices (grazing, permanent cropping systems) as well as to assess resilience of the deforested state to active reforestation practices. For example, the effects of different forest restoration approaches on lowering the resilience of the deforested state could be examined with relatively few modifications to the existing model. Some limitations of our model result also from its semi-spatial character where patch size effects and spatial heterogeneity of agricultural pressure cannot be represented. Integrating this feature through a spatially explicit model is a possible future effort, however with spatially explicit models we lose the capacity to analytically compute stable states. One possibility is to develop both types of models as complementary tools that would allow the separation of spatial and non-spatial effects.

Modeling a forest-agricultural landscape requires certain simplifying assumptions. Nearly all parameters in our models were derived from published data on tropical forest succession in shifting cultivation systems. However, in a few cases (noted in the SI), educated guesses were required to fill gaps in the data. In particular, we were not able to find published data on the recruitment rate for these case studies. For that reason we treated this parameter as an unknown, and modeled system responses to varying recruitment rates. In addition, the meaning of some parameters is difficult to define precisely. While the duration of different successional stages was clearly stated in published articles, it was not always clear whether the stage definitions for different sites were strictly comparable with one another. Further, although succession is clearly a continuous process, we had to approximate it as a stage-based process to accommodate the types of published field data available.

Our study has implications for the management of tropical landscapes under shifting cultivation. It provides further arguments for managers to pay close attention to resilience in addition to agricultural productivity. Whereas maximizing short-term agricultural production is an obvious goal of landscape management, in all examples, our model shows a decline in landscape-level resilience near the maximum level of agricultural output. These findings are particularly significant as they show that sites that appear superficially similar may differ greatly in their risk of state change when managed for optimum agricultural output. Although crop production may be maximized in the short term, intensification of crop cultivation may lead to longer-term declines in both crop and forest productivity. Policy makers and other managers should pay close attention to the long-term sustainability of shifting cultivation. Answering these questions and avoiding unwanted outcomes will require data on how much agricultural pressure the landscape can bear, built upon an understanding of the dynamics of tropical landscapes, as demonstrated by our model.

Our model can be used to estimate levels of external perturbations that can be sustained without triggering a shift from a forested to a deforested state. Parameterizing the model with site-specific data will permit an assessment of the degree to which a given system is approaching a critical threshold—an early warning system. Interventions could range from agricultural extension programs to increase yields to family planning to reduce growth in the demand for food. The response should depend on local ecological and economic characteristics and, therefore, will be site specific. Our results support the view that the persistence of shifting cultivation over several millennia represents a case of socio-ecological resilience that runs counter to the ongoing push toward increasingly intensive agricultural practices in tropical regions.

Finally, our results refocus attention on the resilience of mixed or mosaic forested landscapes, where forests and farmland coexist in a matrix of moderate agricultural land use. Resilience of the forested state can be achieved within a shifting landscape mosaic of croplands and forest patches in different stages of regeneration [47], [48], [49]. These results have significant implications for the controversy surrounding land-sparing as a conservation approach, as agricultural intensification can severely reduce regeneration capacity, which will compromise forest resilience [50], [51], [52]. In particular, the long-term consequences of mechanized and large-scale agricultural land use can reverse the short-term conservation benefits of land sparing. These consequences include land degradation, soil erosion, and contamination of downstream water supplies and groundwater, which will require costly rehabilitation and restoration efforts [53].

In our models, we have striven to capture and analyze the critical features of the dynamics of forest under shifting cultivation. Our model represents only the fundamental processes and their implications for the multistability of the system. The lack of full spatial representation and large time steps required to represent long-term dynamics make these results important for general understanding of shifting cultivation systems but not necessarily for making specific operational decisions. With the complexity of real systems, local management decisions need to include important spatial aspects (e.g. presence of seed stock, seed dispersal, and inter-trophic interactions). However the generality and robustness of our results suggest that multistability (and its resilience implications) is a feature that needs to be taken seriously in any model of forest management under shifting cultivation.

Palm et al. [54] distinguish traditional and sustainable long-fallow shifting cultivation systems from other forms of land use based on slash-and-burn methods that have reduced or no fallow periods, such as conversion of forest to pasture or permanent crops. Our model highlights the role of sufficient fallow periods for soil recovery for the resilience of shifting cultivation systems, and the maintenance of forest within the overall landscape. Under conditions of increasing population pressure, enrichment of fallows through planting or tending economically important trees can be a way to intensify agricultural production while restoring soil fertility and promoting forest resilience in modern shifting cultivation landscapes [17], [55], [56].

## Supporting Information

S1 Fig Forest succession in the absence of agricultural pressure as modeled for six sites. The values here are the product of a numerical solution of forest equations S1–S7 and start from a completely cleared landscape. Panel (a) shows overall forest condition over time for all cases while panel (b) gives a more detailed view of the relative abundance of different forest stages over the course of succession for the Borneo—good soil case.
(TIF)

S2 Fig Agricultural productivity and ecological resilience as a function of agricultural pressure in six sites. Functions are shown for two different values of local recruitment rate, *r* = 0.3 and *r* = 0.5.
(TIF)

S1 Table Values of the forest stage transition parameters (*α*_*i*_) among successional stages in six sites. (DOCX)

S2 Table Agricultural productivity (*β*_*i*_) after clearing forest in stage *i* in six sites. (DOCX)

S1 Supporting Information Additional information about equations and parameters used in the model. (DOCX)
