# Supplementary figures and images for: Resilience and Alternative Stable States of Tropical Forest Landscapes under Shifting Cultivation Regimes
Source: PLoS One. 2015 Sep 25;10(9):e0137497. doi: 10.1371/journal.pone.0137497 (PMC4584006; doi:10.1371/journal.pone.0137497)

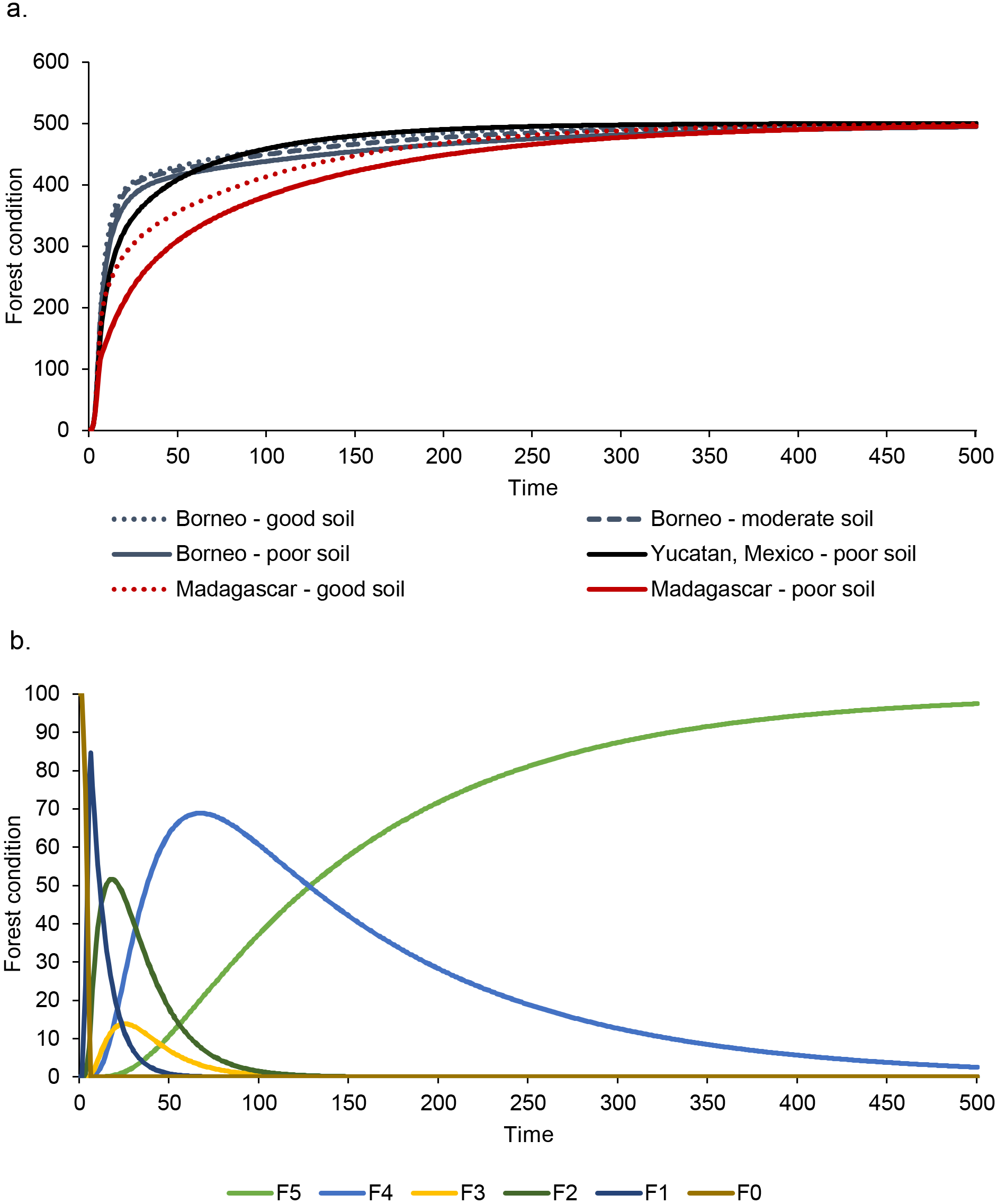

Supplement: S1 Fig — The values here are the product of a numerical solution of forest equations S1–S7 and start from a completely cleared landscape. Panel (a) shows overall forest condition over time for all cases while panel (b) gives a more detailed view of the relative abundance of different forest stages over the course of succession for the Borneo—good soil case. (TIF) [file pone.0137497.s001.tif]

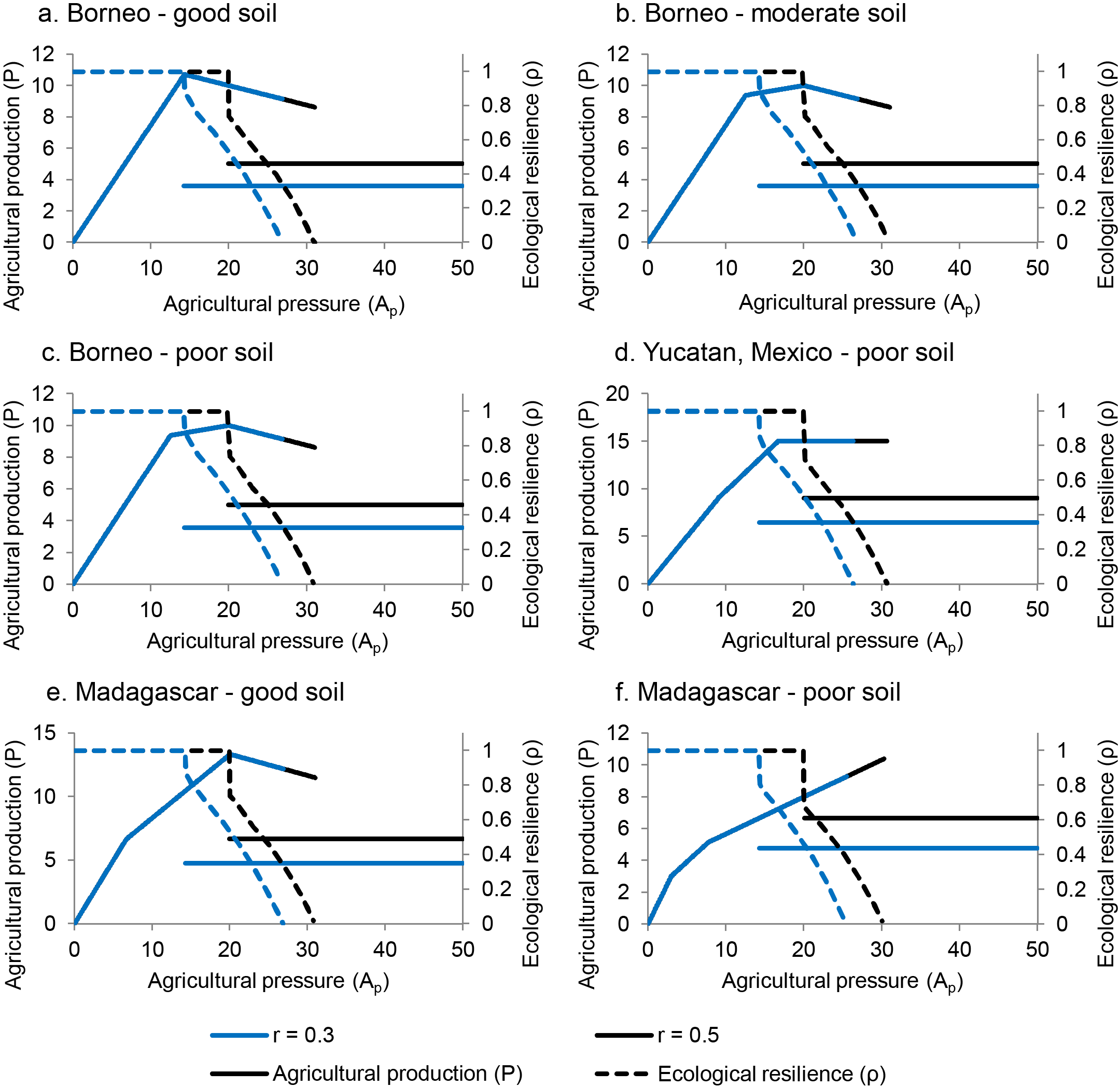

Supplement: S2 Fig — Functions are shown for two different values of local recruitment rate, r = 0.3 and r = 0.5. (TIF) [file pone.0137497.s002.tif]
