# Supplementary material for: Resilience and Alternative Stable States of Tropical Forest Landscapes under Shifting Cultivation Regimes
Source: PLoS One. 2015 Sep 25;10(9):e0137497. doi: 10.1371/journal.pone.0137497 (PMC4584006; doi:10.1371/journal.pone.0137497)
Supplement: S1 Table — (DOCX) [file pone.0137497.s003.docx]

| Transition rate ($\alpha_{i}$) | Borneo | | | Yucatan, | Madagascar | |
| --- | --- | --- | --- | --- | --- | --- |
|  | Good soil | Moderate soil | Poor soil | Poor soil | Good soil | Poor soil |
|  | 0.500 | 0.500 | 0.500 | 0.333 | 0.500 | 0.100 |
|  | 0.500 | 0.333 | 0.333 | 0.200 | 0.100 | 0.050 |
|  | 0.167 | 0.167 | 0.100 | 0.050 | 0.020 | 0.017 |
|  | 0.010 | 0.008 | 0.006 | 0.014 | 0.010 | 0.008 |
