# Supplementary material for: Resilience and Alternative Stable States of Tropical Forest Landscapes under Shifting Cultivation Regimes
Source: PLoS One. 2015 Sep 25;10(9):e0137497. doi: 10.1371/journal.pone.0137497 (PMC4584006; doi:10.1371/journal.pone.0137497)
Supplement: S2 Table — (DOCX) [file pone.0137497.s004.docx]

| Agricultural productivity () | Borneo | | | Yucatan, Mexico | Madagascar | |
| --- | --- | --- | --- | --- | --- | --- |
|  | Good soil | Moderate soil | Poor soil | Poor soil | Good soil | Poor soil |
|  | 0 | 0 | 0 | 0 | 0 | 0 |
|  | 0.25 | 0.25 | 0.25 | 0.45 | 0.333 | 0.333 |
|  | 0.5 | 0.5 | 0.5 | 0.9 | 0.667 | 0.667 |
|  | 0.75 | 0.75 | 0.75 | 1 | 1 | 1 |
|  | 1 | 1 | 1 | 1 | 1 | 1 |
|  | 1 | 1 | 1 | 1 | 1 | 1 |
