# Supplementary material for: Resilience and Alternative Stable States of Tropical Forest Landscapes under Shifting Cultivation Regimes
Source: PLoS One. 2015 Sep 25;10(9):e0137497. doi: 10.1371/journal.pone.0137497 (PMC4584006; doi:10.1371/journal.pone.0137497)
Supplement: S1 Supporting Information — (DOCX) [file pone.0137497.s005.docx]

**Forest equations**

Forest area is the sum of all forest stages *-* . It does not include fallow land (*)*:

Land used for agriculture in the previous time step becomes fallow and new patches of forest are cleared for agricultural use:

(S1)

Land used for agriculture in the previous time step becomes fallow and some of the fallow land regenerates into forest:

(S2)

The change in area in stage depends on the balance between regeneration from fallow land, clearing of land in stage for agriculture, and maturation of stage forest into .

(S3)

Changes in area of stages to have the same component as ; some of the land may be converted for agriculture, some of the forest from the previous stage may mature, and some of the forest may mature into the next stage.

(S4)

(S5)

(S6)

Stage forest also results from maturation of the previous stage, but can only be lost by conversion to agriculture.

(S7)

Figure S1 shows example numerical solutions of equations [S1-S7].

**Parameterization**

We parameterized this model with all published examples we could find in the peer-reviewed literature that had sufficient information on forest succession in the context of shifting cultivation. These amounted to six sites in three countries, Mexico, Borneo and Madagascar. We have implemented combinations of parameters values based on traditional ecological knowledge of Mayans (Mexico, 1 case; Barrera-Bassols and Toledo, 2005), Benuaq Dayak (Borneo, 3 cases corresponding to different values of soil quality; Abdoellah et al., 1993) and Betsimisaraka (Madagascar, 2 cases corresponding to different values of soil quality; Styger et al., 2007). The values of transition rates () were chosen based on published site-specific estimates of the time forests require to reach specific stages.

Forest productivity weights () were chosen as follows. For the Mayan case, the values were based on the fallow length times found in Lawrence et al (2010). The range of fallow lengths (6-9 years) for the Yucatan puts the full recovery of soil fertility near the beginning of stage F2. Thus, we assigned F2 a nearly fully recovered value (0.9) and put F1 at 0.45, half way between 0 and 0.9. Later stages were all assigned a value of 1 (full fertility). For Benuaq Dayak cases, the values are based on the fallow length times found in Lawrence et al (2010). The fallow length puts full soil recovery near the beginning of stage F4, so we increased fertility linearly (in forest stage units, not time units) from F0 to F4. For the Betsimisaraka cases, according to Styger et al. (2007), a patch is ready to be cultivated again once it has reached the “*Savoka Matoy*” stage (F3). Thus we assigned a value of 1 to that stage and higher. The values for the intervening stages were assigned as equally spaced steps.

Forest condition weights () were chosen linearly from 1 to 5 to reflect a monotonically increasing value of the forest with successional stage. The increasing value of with forest age reflects the greater diversity of species present in these forests, particularly late successional species, and their contribution to succession in nearby abandoned agricultural fields. While this value clearly increases with forest age, there is a lack of empirical data on exactly how this changes with forest age, so we assume a linear increase for simplicity.

**References**

Abdoellah O, Lahjie AB, Wangsadidjaja SS. Communities and forest management in East Kalimantan: Pathways to environmental Sustainability. In: Poffenberger M, McGean B, editors. Center for Southeast Asia Studies Research Network Report Number 3. Berkeley: University of California; 1993.

Barrera-Bassols N, Toledo VM. Ethnoecology of the Yucatec Maya: Symbolism, knowledge and management of natural resources. **J. Lat. Am. Geogr.** 2005; 4:9-41.

Lawrence D, Radel C, Tully K, Schmook B, Schneider L. Untangling a decline in tropical forest resilience: Constraints on the sustainability of shifting cultivation across the globe. Biotropica. 2010; 42.1: 21-30.

Styger E, Rakotondramasy HM, Pfeffer, MJ, Fernandes ECM, Bates DM. Influence of slash-and-burn farming practices on fallow succession and land degradation in the rainforest region of Madagascar. Agric. Ecosyst. Environ. 2007; 119:257-269.

**Closed form solutions for stable states**

For there exists a stable stationary “deforested” state, characterized by:

; ,

,

;

,

(S8)

Another stable stationary state may exist depending on the value of $A$:

I. For $A_{p}\leq f_{3}\Rightarrow f_{1}^{A}=f_{2}^{A}=f_{4}^{A}=f_{5}^{A}=0$,:

, , , , , (S9)

It exists if: .

II. For , :

, , , , , , (S10)

exists if

,

III. For , :

, , , , (S11)

exists if

, ,

IV. For :

another stationary state exists only for and then: , .

There exist also unstable stationary states, that will not be considered here.

It is worth noticing that for only one stable stationary state exists (“forested”), while for again only one stationary stable state exists, but now it is “deforested” state

**Closed form solution for equilibrium agricultural production as function of agricultural pressure**

Forest condition (stationary value) is a monotonically decreasing function of the agricultural pressure. On the other hand, agricultural production is an increasing function for the low values of the agricultural pressure, approaching maximum value and then becoming decreasing function for higher values of agricultural pressure. Specifically, it is always an increasing function for :

. (S12)

It is either increasing or decreasing function in the range: :

. (S13)

Thus, it is increasing within this range for values of parameters fulfilling:

. (S14)

Finally, for it is an increasing function for:

, (S15)

or decreasing otherwise.

Thus the maximum production equals to:

. (S16)
